# Visualization of cyclic nucleotide dynamics in neurons

**DOI:** 10.3389/fncel.2014.00395

**Published:** 2014-12-04

**Authors:** Kirill Gorshkov, Jin Zhang

**Affiliations:** Laboratory of Dr. Jin Zhang, Department of Pharmacology and Molecular Sciences, Johns Hopkins University School of MedicineBaltimore, Maryland, USA

**Keywords:** biosensor, neuron, FRET, fluorescence, signaling, cyclic nucleotide, cAMP, cGMP

## Abstract

The second messengers cyclic adenosine monophosphate (cAMP) and cyclic guanosine monophosphate (cGMP) transduce many neuromodulatory signals from hormones and neurotransmitters into specific functional outputs. Their production, degradation and signaling are spatiotemporally regulated to achieve high specificity in signal transduction. The development of genetically encodable fluorescent biosensors has provided researchers with useful tools to study these versatile second messengers and their downstream effectors with unparalleled spatial and temporal resolution in cultured cells and living animals. In this review, we introduce the general design of these fluorescent biosensors and describe several of them in more detail. Then we discuss a few examples of using cyclic nucleotide fluorescent biosensors to study regulation of neuronal function and finish with a discussion of advances in the field. Although there has been significant progress made in understanding how the specific signaling of cyclic nucleotide second messengers is achieved, the mechanistic details in complex cell types like neurons are only just beginning to surface. Current and future fluorescent protein reporters will be essential to elucidate the role of cyclic nucleotide signaling dynamics in the functions of individual neurons and their networks.

## Introduction

The cyclic nucleotides cyclic adenosine monophosphate (cAMP) and cyclic guanosine monophosphate (cGMP) are ubiquitous second messengers present in most cell types. Within the brain, cyclic nucleotides transduce neuromodulatory signals into functional outputs for individual neurons leading to changes in neural networks themselves or their function. The importance of cyclic nucleotide signaling pathways is well appreciated in the field of clinical neuroscience and psychiatry, with many drugs targeting the G-protein coupled receptors to modulate neuronal activity (Lim, 2007; Taly, 2013). The effects of cAMP and cGMP signaling range from regulating neuronal differentiation and growth to axonal guidance and modulation of neuronal excitability. To accomplish this, cyclic nucleotides are coupled to many downstream effectors (Antoni, 2000). cAMP, the prototypical cyclic nucleotide, transduces G-protein signals to activate protein kinase A (PKA) and exchange protein activated by cAMP (Epac). cGMP, on the other hand, relays signals from nitric oxide to activate protein kinase G (PKG). Phosphodiesterase (PDE), the enzyme that degrades cGMP, can be a cGMP effector with its activity modulated by cGMP binding to regulatory domains forming feedback loops (Conti and Richter, 2014). Both cyclic nucleotides also activate cyclic nucleotide gated ion channels (Rich et al., 2014).

The idea of compartmentalized signaling is an essential component to the cyclic nucleotide signaling model. To elicit their diverse functional effects in a highly specific manner, cAMP and cGMP signaling is thought to be spatially compartmentalized (Zaccolo and Stangherlin, 2014). The levels of cyclic nucleotides and the activities of downstream effectors are not uniform throughout the cell, but instead form specific nanodomains or microdomains inside the cell. The spatial compartmentation is achieved, at least partially, by strict regulation of cyclic nucleotide production and degradation. cAMP is produced by adenylyl cyclase (AC) and cGMP is produced by guanylyl cyclase (GC). These enzymes are located within the plasma membrane as transmembrane proteins, or within the cytoplasm or organelles as soluble forms of the enzyme. The degradation of cAMP and cGMP is carried out by PDEs which have specificity for cAMP, cGMP, or both. PDEs have been shown to function as cAMP and cGMP sinks to help maintain these microdomains (Terrin et al., 2006; Biswas et al., 2008). The tight spatiotemporal regulation of cAMP signaling is achieved with the help of the A-kinase anchoring protein (Esseltine and Scott, 2013), which assembles signaling complexes consisting of members of the cAMP/PKA signaling pathway like ACs, PDEs, PKA and its substrate, and other effectors. These signalosomes, which can be found throughout various compartments including the plasma membrane, the cytosol and the nucleus, have been shown to play important roles in achieving functional specificity of the cAMP/PKA pathway. In addition to biochemical regulation, the structural properties of cells can also affect the signaling dynamics of second messengers (Castro et al., 2010; Neves, 2012).

Relatively recent advances in fluorescent biosensor technology allow researchers to track the dynamics of cyclic nucleotides and their effectors in living neurons and brain tissue. Here we will describe several genetically encoded fluorescent biosensors for monitoring cyclic nucleotide dynamics. Then we will focus on a few studies that demonstrate their implementation in living neurons for the purpose of understanding how cyclic nucleotides impact neuronal function. We will end with a discussion of novel tools and modeling efforts that help us understand neuronal cyclic nucleotide dynamics.

## Genetically encoded fluorescent biosensors

**Figure 1:**
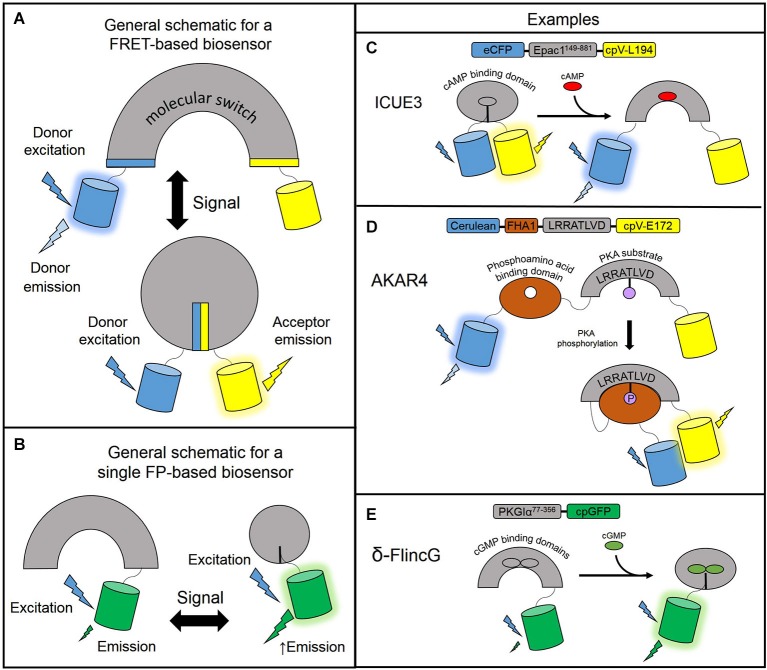
**Designs of genetically encodable biosensors**. **(A)** General design of a FRET-based biosensor consisting of a sensing unit which acts as a molecular switch to change the distance or orientation of the fluorescent protein reporting unit. In a low FRET situation, the donor fluorescent protein is excited and emits light at its own wavelength. In the high FRET situation, donor excitation allows for resonance energy transfer to the acceptor fluorescent protein which emits light at its own wavelength. **(B)** General design of a single fluorescent protein-based biosensor. The sensing unit transduces a signal via a conformational change to the linked fluorescent protein reporting unit which undergoes its own conformational change and modulates fluorescence intensity. A circularly permuted GFP is often used to enhance the change in fluorescence. **(C)** ICUE3 consists of an Epac1^149–881^ sensing unit flanked by an ECFP donor and a cpV-L194 acceptor reporting unit. Upon binding cAMP, the sensor switches from a high FRET to a low FRET conformation. **(D)** AKAR4 contains a sensing unit consisting of a FHA1 phosphoamino acid binding domain and a substrate peptide. The reporting unit is comprised of a Cerulean donor and cpV-E172 acceptor reporting unit. When PKA activity is high, the substrate peptide is phosphorylated and binds the FHA1 domain to induce FRET. **(E)** δ-FlincG utilizes a PKG1α^77–356^ sensing unit linked to a single cpGFP reporting unit. Upon binding cGMP, the fluorescence intensity of cpGFP increases.

The genetically encoded fluorescent biosensors described in the following sections allow for the continuous monitoring of free cyclic nucleotides with high spatiotemporal resolution. These biosensors are engineered based on a general design: a sensing unit to detect the change in free cyclic nucleotide concentration and a reporting unit to convert the biochemical change into a fluorescent readout. The sensing unit for cyclic nucleotide biosensors is one or more cyclic nucleotide binding domains (CNBD). The reporting unit can be made up of two fluorescent proteins flanking the sensing unit as is the typical arrangement for fluorescence resonance energy transfer (FRET)-based biosensors. In FRET, energy is transferred non-radiatively from an excited donor molecule to an acceptor molecule. For a fixed FRET donor-acceptor pair, the efficiency of FRET is dependent on the distance and orientation of the two fluorophores. In a FRET-based biosensor, cyclic nucleotide binding induces conformational changes of the CNBD sensing unit, which acts as a molecular switch to change the physical separation or orientation between the fluorescent protein pair resulting in a change in FRET (Figure 1A). In intensity-based biosensors, on the other hand, the reporting unit can be a single fluorescent protein. In this case, conformational changes in the sensing unit are translated into changes in fluorescence intensity (Figure 1B). Different sensors vary in their CNBD sensing units and fluorescent protein reporting units. For a thorough overview of fluorescence and fluorescent proteins used in many different types of biosensors, please refer to the reviews by Sample et al. (2009); Newman et al. (2011). The following subsections describe the various designs of many cAMP and cGMP reporters. Please refer to Table 1 for a detailed list of the biosensors described here.

**Table 1:** **A detailed list of biosensors currently available for cAMP, PKA, and cGMP**.

Sensor name	Sensing domain	Design	EC_50_ (cAMP)	Response	Comments	Reference
**cAMP**
FICRhR	PKA catalytic and RIIβ subunits	Multimeric	0.09 μM	FRET ↓	Chemically labeled, high affinity	Adams et al. (1991)
	*Genetically encoded sensors*							
RII-CFP; C-YFP	As above	Multimeric	~0.3 μM	FRET ↓	Original version contained EBFP/EGFP, newest version has a 20 aa linker between CFP and Rll	Zaccolo and Pozzan (2002), Mongillo et al. (2004), Lissandron et al. (2005)
PKA-camps	PKA RIIβ subunit	Single Chain	1.9 μM	FRET ↓	Less robust response than Epac-camps	Nikolaev et al. (2004)
Epac-camps family	Epac 1/2 CNBD	Single Chain	Epac1-camps—2.4 μM, Epac2-camps—0.9 μM, Epac2-camps300—~0.3 μM	FRET ↓	Epac2-camps300 has K405E mutation, range of relatively high affinities	Nikolaev et al. (2004), Norris et al. (2009)
ICUE Family	Full length or truncated Epac	Single Chain	ICUE2/3—~12.5 μM	FRET ↓	cpV-L194 replaces Citrine in ICUE3, doubles the dynamic range	DiPilato et al. (2004), Violin et al. (2008), DiPilato and Zhang (2009)
^T^Epac^VV^	Epac Δ DEP, catalytically dead (CD)	Single Chain	~14 μM for CFP-YFP version	FRET ↓	Lower affinity, T781A and F782A catalytically dead mutations, mTurquoiseΔ and tandem cpV- E173-Venus reporting unit	Ponsioen et al. (2004), Klarenbeek et al. (2011)
Epac-S^H150^	Epacl Δ (DEP, CD) Q270E	Single Chain	4.0 μM	FRET ↓	Higher affinity, mTurquoise2 and cpCitrine, large dynamic range	Polito et al. (2013)
HCN2-camps	HCN2 CNBD	Single Chain	6.0 μM	FRET ↓	Based on CNGC	Nikolaev et al. (2006a)
Nanolantem-camps	Epac 1^170–327^, Q270E	Single Chain	1.6 μM	Luminescence ↑	Uses VenusΔC10 and split Rluc8ΔN3, no excitation light reguired, low absolute intensity	Saito et al. (2012)
**PKA**
AKAR family	FHA1 PAABD and PKA substrate	Single Chain	NA	FRET ↑	Cerulean and cpV-E172 in AKAR4, very bright, fast kinetics, amplifies cAMP signal through kinase activity	Zhang et al. (2001), Allen and Zhang (2006), Depry et al. (2011)
^Aq^AKAR^Cit^	Same as AKAR	Single Chain	NA	FRET ↑	Uses Aquamarine and cpCitrine, stable to environmental pH	Erard et al. (2013)
GAkdY family	Same as AKAR	Single Chain	NA	Fluorescence ↑	Conformationally senstive GFP variant incorporated into AKAR, single wavelength probe, two-photon imaging of dendrites and spines	Bonnot et al. (2014)
**Dual Specificity Probe**
ICUEPID	As above plus Epac 1	Single Chain	NA	For PKA (CFP-RFP) FRET ↑ For cAMP (RFP-YFP) FRET↓	Dual specificity for cAMP/PKA for co-imaging using single construct	Ni et al. (2011)
**Epac**
CFP-Epac2-YFP	Full length Epac	Single Chain	NA	FRET ↓	Reports on [cAMP] as well as Epac dynamics	Zhang et al. (2009), Herbst et al. (2011)
**cGMP**
CGY-Del1	PKG1α CNBD	Single Chain	20 nM	FRET ↑	cGMP/cAMP selectivity = 7.6	Sato et al. (2000), Nikolaev et al. (2006b)
Cygnet family	PKG1α CNBD	Single Chain	cygnet-1 = 1.5 μM, cygnet-2 = 1.9 μM	FRET ↓	cGMP/cAMP selectivity cygnet-2.1 >600	Honda et al. (2001)
cGES family	PDE2/5 GAF CNBD	Single Chain	cGES-DE2 = 0.9 μM, cGES-DE5 = 1.5 μM. redcGES-DE5 = 40 nM	FRET ↑	cGMP/cAMP selectivity cGES-DE5 = 420	Nikolaev et al. (2006b), Niino et al. (2009)
cGi family	tandem PKGIα CNBD	Single Chain	cGi500 = 0.5 μM, cGi3000 = 3.0 μM, cGi6000 = 6.0 μM	FRET ↑	fast kinetics with high selectivity	Russwurm et al. (2007)
FlincG family	truncated PKG1α	Single Chain	δ-FlincG = 0.15 μM	Intensity ↑	cGMP/cAMP selectivity = 1140, 30, 280 for α, β, δ, respectively	Nausch et al. (2008)
Cygnus	PDE5 GAF-A CNBD	Single Chain	1.0 μM	Intensity ↑	Uses mTagBFP and dark YFP sREACH, cGMP/cAMP selectivity >400, useful for multi-parameter imaging	Niino et al. (2010)

### Fluorescent indicators of cAMP

#### cAMP reporter based on dissociation of PKA holoenzyme

For the purposes of tracking cAMP, researchers have been developing molecular biosensors for the past several decades. The first of such biosensors called FlCRhR (**Fl**uorescein-labeled PKA **C**atalytic subunit and **Rh**odamine-labeled **R**egulatory subunit) was based on chemically labeled regulatory and catalytic subunits of the PKA holoenzyme (Adams et al., 1991). Upon cAMP binding the catalytic and regulatory subunits dissociate producing a change in FRET. This labeled holoenzyme was microinjected into living cells and imaged under widefield microscopy. A genetically encodable version was generated allowing for expression in a wide array of cell types, thereby expanding the scope of the application (Zaccolo et al., 2000). The fusion protein contained an 11 amino acid linker between the PKA regulatory subunit and the fluorescent proteins (RII-EBFP; C-GFP^S65T^). A newer version was generated using CFP fused to the RII regulatory domain and YFP fused to the catalytic domain (Zaccolo and Pozzan, 2002; Mongillo et al., 2004). By using molecular dynamics simulations, this design was further improved with the introduction of a longer, more rigid 20 amino acid linker (Lissandron et al., 2005). Live-cell fluorescent lifetime and acceptor sensitized measurements of this sensor showed a doubling of the dynamic range.

#### Single-chain FRET-based cAMP indicators

Multimeric biosensors like RII-CFP/C-YFP require equal expression of both subunits to form the PKA tetramer. In addition, there can be interactions with endogenous PKA subunits which do not make contributions to the FRET response. On the other hand, single chain biosensors offer ease of use and targeting to subcellular locations, increasing the spatial resolution offered by FRET-based biosensors. In 2004, three groups independently developed single-chain cAMP FRET sensors using the CNBD from Epac, a Rap1 guanine nucleotide exchange factor, or PKA. The reported affinities for cAMP range from ~0.3 to 50 μM. However, we note that experimental variations such as biosensor concentrations when determining EC_50_s could largely affect the resulting values. We include the experimentally determined EC_50_s in Table 1 but these values may not accurately reflect the differences in biosensor affinities.

In Epac1-camps, the cAMP binding domain of the Epac1 protein (Epac1^157–316^) was flanked by an N-terminal CFP and a C-terminal YFP (Nikolaev et al., 2004). Epac2-camps used the cAMP binding domain B from Epac2 (Epac2B^284–443^) instead. Both biosensors generated a decrease in the yellow to cyan emission ratio upon binding cAMP, indicating a decrease in FRET. In an attempt to generate a more sensitive cAMP probe called Epac2-camps300, Norris et al. introduced a K405E mutation into Epac2-camps that decreased the EC_50_ from ~0.9 μM to ~0.3 μM (Norris et al., 2009). Nikolaev et al. also generated a single chain PKA-camps using a portion the PKA regulatory βII subunit (RIIβ) sandwiched between ECFP and EYFP (Nikolaev et al., 2004). PKA-camps incorporated the cAMP binding domain B from amino acids 264–403 of RIIβ as the sensing unit. Upon binding cAMP, RIIβ undergoes a conformational change conducive to a change in FRET. These single CNBD containing biosensors are the smallest cAMP probes currently available.

A series of Epac based reporters called ICUE (**I**ndicator of **c**AMP **U**sing **E**pac) have been developed in parallel. First in the series, ICUE1 contained full-length Epac1 sandwiched between ECFP and the YFP variant Citrine (DiPilato et al., 2004). Like Epac1-camps and Epac2-camps, ICUE also responds to cAMP with a decrease in the yellow to cyan emission ratio. ICUE2, an improved version of ICUE1, has an EC_50_ of ~12.5 μM and contains a N-terminally truncated Epac1 protein (Epac1^149–881^) (Violin et al., 2008). This biosensor showed improvement in localization over ICUE1 due to removal of a membrane and mitochondria targeting sequence located at the N-terminus. More recently, we developed ICUE3 with an increased dynamic range by changing the FRET acceptor Citrine to a circularly permuted Venus at lysine 194 (cpV-L194; DiPilato and Zhang, 2009; Figure 1C). The large dynamic range of ICUE3 (~100% emission ratio change) makes it suitable for subcellular targeting for detecting local cAMP changes (e.g., plasma membrane and nucleus (Sample et al., 2012), sarcoplasmic reticulum (Liu et al., 2012), primary cilia (Marley et al., 2013)) as addition of subcellular localization tags sometimes leads to decreased response amplitudes.

The Jalink lab developed a similar biosensor CFP-Epac(δDEP-CD)-YFP using Epac1^149–881^ flanked by an amino-terminal CFP and carboxy-terminal YFP (Ponsioen et al., 2004). Mutations T781A and F782A were introduced to generate a catalytically dead (CD) Epac1. The lab has recently developed newer versions, some of which are useful for fluorescence lifetime imaging FRET (FLIM-FRET) measurements (Klarenbeek et al., 2011; Polito et al., 2013). Named Epac-S^H150^, the newest version in this series incorporated the Q270E mutation (Dao et al., 2006) to increase the affinity for cAMP (EC_50_ 4 μM) and used mTurquoise2 as the FRET donor and a single circularly-permuted Citrine as the FRET acceptor (Polito et al., 2013).

In some cases, the concentration of cAMP in a cell can saturate a highly sensitive reporter. To accommodate large fluctuations in signaling, Nikolaev et al. used the hyperpolarization-sensitive cyclic nucleotide gated channel 2 (HCN2) to generate HCN2-camps and image large changes in cAMP concentration in cardiomyocytes (Nikolaev et al., 2006a). In the context of probes like PKA-camps, Epac-camps, and CFP-RII/C-YFP, this probe exhibited a lower affinity for cAMP (EC_50_ ~6 μM) and provides another tool to monitor cAMP in cells with larger basal or fluctuating concentrations.

#### Indicators of downstream cAMP effectors

Neuromodulatory signals can be transduced by cAMP and cGMP through their downstream target kinases PKA and PKG, respectively (Giese and Mizuno, 2013). Although there are no biosensors yet available for PKG activity, the A-kinase activity reporter (AKAR) can report on the kinase activity of PKA (Zhang et al., 2001). AKAR uses a molecular switch consisting of a phosphoamino acid binding domain linked to a PKA-specific substrate sequence, flanked by CFP and YFP. PKA phosphorylation of its substrate induces binding of the phosphorylated substrate to the phosphoamino acid binding domain, leading to an increase in FRET. AKAR1 displayed an irreversible FRET response which prevented continuous monitoring of PKA dynamics. This was presumably due to the high affinity of the 14-3-3 binding domain for the substrate as tight binding may prevent phosphatases from dephosphorylating the substrate and reversing the FRET response. This was overcome by the generation of AKAR2 which utilized the lower binding affinity forkhead-associated domain 1 (FHA1) and exhibited a reversible FRET response (Zhang et al., 2005). The kinetics of AKAR2 were improved in AKAR2.2 by replacing the dimeric forms of ECFP and Citrine with versions that resist dimerization. The dynamic range was doubled in AKAR3 by replacing the YFP acceptor in AKAR2 with cpV-E172 (Allen and Zhang, 2006). The dynamic range of AKAR was further enhanced with latest version, AKAR4, by replacing ECFP with Cerulean (Depry et al., 2011; Figure 1D). Due to the amplification of the cAMP signal by PKA phosphorylation activity, PKA activity reporters may be able to detect signals not picked up by cAMP binding probes. Recently, a modified AKAR probe named *^Aq^*AKAR*^Cit^*, was generated by replacing Cerulean with Aquamarine, a newly engineered CFP variant, which has mutations T65S and H148G (Erard et al., 2013). These modifications to ECFP increased its photophysical properties and reduced its environmental sensitivity to low pH.

The modular design of FRET-based biosensors allows researchers to couple the same sensing unit to different reporting units. For example, red fluorescent protein (RFP) can act as an acceptor for both CFP and YFP. ICUE2 was redesigned as a YFP-RFP FRET sensor (YR-ICUE) and co-imaged with a CFP-RFP-AKAR (CR-AKAR) to analyze the temporal relationship between cAMP and PKA signaling following receptor activation in HEK-293 cells (Aye-Han et al., 2012). Utilizing the CFP-RFP and YFP-RFP based biosensors for co-imaging is a simple technique to monitor two biochemical events in parallel and only requires the addition of an RFP emission filter to the imaging setup. With the shared RFP receptor, these two biosensors were further combined to generate the single-chain dual-specificity probe ICUEPID which can sense both cAMP and PKA simultaneously (Ni et al., 2011). It utilizes CFP and YFP donors and a single RFP acceptor. The PKA activity sensing unit is flanked by CFP and RFP while the cAMP sensing unit is flanked by RFP and YFP. This sensor proved useful in Min6 pancreatic β-cells to detect synchronized oscillations of cAMP and PKA within the same subcellular location.

A single-wavelength PKA activity sensor was designed by utilizing a conformation sensitive GFP variant and combining it with the sensing unit of AKAR2 to generate GAkdY (Bonnot et al., 2014). PKA phosphorylation of the substrate changes the conformation of the probe and modulates GFP fluorescence intensity and lifetime. Single color activity sensors such as this probe provide a way to image multiple kinase activities with single excitation wavelengths for each sensor. Three of these sensors were incorporated into Sindbis viral vectors and expressed in brain slices to visualize PKA dynamics in pyramidal cell bodies, thin dendrites, and dendritic spines using two-photon microscopy.

Indicators of enzyme activation typically have sensing domains comprised of the full length endogenous protein. Epac1 and Epac2 share similar activation mechanisms whereby cAMP binding relieves the steric block of the regulatory domain on the Rap1 binding catalytic site by inducing a conformational change in the regulatory domain hinge helix (Selvaratnam et al., 2012). In this context, ICUE1 is also an Epac1 activation probe. A FRET reporter for Epac2 activation was generated by sandwiching full length Epac2 between ECFP and EYFP (Zhang et al., 2009). A brighter Epac2 activation reporter is also available containing Cerulean and Venus, variants of CFP and YFP, respectively (Herbst et al., 2011).

### Fluorescent indictors of cGMP

Cyclic guanosine monophosphate probes, like cAMP probes, report the presence of cGMP by using a CNBD derived from PKG or cGMP-specific PDEs. Because the concentration of cGMP in neurons is lower than cAMP, the probes must be highly sensitive and specific in order to provide a high signal to noise ratio and large dynamic range.

#### Single-chain FRET-based indicators of cGMP

The cygnet series of cGMP reporters was developed by the Dostmann lab and utilized both CNBDs from PKG1α as a sensing unit that responds to cGMP (Honda et al., 2001). Therefore, each molecule of cygnet binds two molecules of cGMP. In cygnet-1 (**cy**clic **G**MP i**n**dicator using **e**nergy **t**ransfer), the first 77 amino acids of PKG1α (PKG1αΔ^1–77^) were truncated. PKG1αΔ^1–77^ was flanked by a reporting unit comprised of ECFP and EYFP at the N- and C- termini, respectively. Cygnet-2, the catalytically inactive version of cygnet-1, was generated by introducing mutation T516A to PKG1αΔ^1–77^. The pH-insensitive EYFP variant Citrine was used to replace YFP in cygnet-2 to generate cygnet-2.1. cGMP binding to the cygnet reporters induces a decrease in FRET. Cygnet-1 and cygnet-2 have an affinity of 1.5 μM and 1.9 μM for cGMP, respectively, although endogenous PKG1α has a cGMP affinity of ~100 nM. It is conceivable that fusion of the fluorescent proteins or the Δ1–77 N-terminal truncation and catalytic site mutations affected their cGMP binding affinity. Around the same time, Sato et al. generated a similar PKG1α based probe called CGY-Del1 which had an N-terminal truncation of the first 47 amino acids (Sato et al., 2000). cGMP binding induces an increase in FRET in this probe.

Nikolaev et al. developed smaller probes utilizing a single CNBD from PKG1α^231–350^, PDE2^392–525^ and PDE5^154–308^ (Nikolaev et al., 2006b). Their efforts culminated in the generation of three sensors, cGES-GKIB (for cGMP energy transfer sensor derived from GKI-B (PKGI) site), cGES-DE2 (derived from PDE2A), and cGES-DE5 (derived from PDE5A). All three FRET sensors had reporting units consisting of EYFP and ECFP at the N- and C- termini, respectively. Interestingly, cGES-GKIB exhibited a cGMP-dependent decrease in FRET whereas cGES-DE2/5 exhibited a cGMP-dependent increase in FRET. These three probes all exhibited strong FRET responses, but cGES-DE5 containing the GAF-A domain of PDE5A had a ~400–600 fold greater selectivity for cGMP:cAMP making it the preferred cGMP sensor for live-cell applications. More recently, a red version of cGES-DE5 was generated for co-imaging experiments with CFP-YFP sensors by using the GFP variant T-SapphireCΔ11 and RFP dimer2 (Niino et al., 2009). Surprisingly, switching the CFP-YFP to GFP-RFP variants increased the affinity of cGES-DE5. The large enhancement in the probe affinity (EC_50_ ~ 40 nM) makes it potentially suitable for detecting low cGMP concentrations but may also be confounded by variations in experimental conditions when determining the EC_50_. The probe was used to develop a method for simultaneous measurements of signaling activities by using a single excitation light that excites both T-sapphire and CFP, four channel detection and linear unmixing.

Russwurm and colleagues generated a series of FRET based cGMP sensors in order to achieve faster kinetics and provide an array of probes with a range of affinities (Russwurm et al., 2007). These sensors used the tandem CNBD domains from PKGIα as their sensing unit. Beginning with the indicator construct CFP-PKGIα^79–336^-YFP, they elongated the N- and C- termini of PKGIα^79–336^ and screened for constructs based on cGMP affinity and the FRET response amplitude. Using this approach, they generated a series of cGMP biosensors cGi-500 (EC_50_ = 500 nM), cGi-3000 (EC_50_ = 3.0 μM), and cGi-6000 (EC_50_ = 6.0 μM).

#### Single-FP based cGMP sensors

Nausch et al. generated a line of biosensors called fluorescent indicators of cGMP (FlincG; Nausch et al., 2008). The reporting unit of FlincG contains a single circularly-permuted EGFP (cpEGFP) molecule. In this series, complete or truncated cGMP binding regulatory domains from PKG1 were used to construct the sensing unit. First in line, α-FlincG used the complete regulatory domain of PKG1α^1–356^. Second, β-FlincG contained the complete regulatory domain from PKG1β, which has an activation constant of 1.0–1.8 μM compared to 75 nM for PKG1α, fused to the N-terminus of cpEGFP. PKGIβ has a completely different N-terminus than PKG1α, which highlights the importance of the N-terminus for cGMP binding affinity. Lastly, the researchers removed the entire N-terminal domain, the first 77 amino acids of PKG1α, to generate δ-FlincG (Figure 1E). This decreases the *K_D_* of PKG1αΔ^1–77^ to ~170 nM. Because δ-FlincG had a superior dynamic range and retained nanomolar affinity for cGMP in living cells, it was chosen as the preferred single-GFP linked cGMP biosensor for further characterization and application. Single-color sensors with adequate spectral separation allow for multi-parameter imaging of interacting molecules in complex signal transduction networks. In addition to the green cGMP sensor described above, a blue single-color cGMP sensor named Cygnus was developed by using a blue fluorescent protein (BFP) and a dark fluorescent protein acceptor (Niino et al., 2010). This biosensor was generated by sandwiching the GAF-A domain of PDE5 between mTagBFP and the quenching acceptor YFP sREACH. Cygnus was used to demonstrate cGMP imaging in rat hippocampal neurons and triple parameter imaging of Ca^2+^, cAMP, and cGMP in HEK-293T cells.

## Application of cyclic nucleotide biosensors to study neuronal systems

The following section highlights a few studies that utilize cyclic nucleotide biosensors in investigating neuronal polarization, axon guidance and growth, signaling, and plasticity.

### Polarization

Cyclic adenosine monophosphate and PKA are one of the few bona fide axon determinants that play a critical role in axon polarization (Cheng and Poo, 2012). In a recent study, Shelly et al. investigated the contributions of cAMP and cGMP to the process of axon and dendrite formation of early stage hippocampal neurons in isolated cultures. Given that cAMP and cGMP exerted opposing actions in other cell systems, it was possible that they played some role in the differentiation of neuronal processes to form distinct compartments. It was discovered that neurites exposed to cAMP have a high probability of differentiating into axons and those exposed to cGMP become dendrites (Shelly et al., 2010). But how are these processes coordinated in a single cell to ensure that only one neurite becomes the axon? Using the fluorescent biosensors ICUE and cGES-DE5 the researchers examined the effects of locally stimulating a single neurite with a glass bead soaked in cAMP agonist or cGMP analog. Local elevation of cAMP in one of the neurites resulted in a decrease of cAMP and increase of cGMP at the other neurites. Locally elevating cGMP only decreased cAMP at the stimulated neurite and did not exhibit long range inhibition of cGMP. They concluded that local and long range reciprocal regulation of cAMP and cGMP ensures the development of a single axon and multiple dendrites, although the exact mechanism of long range inhibition remains to be elucidated.

The question still stands as to which endogenous factors act through cAMP and cGMP to induce a single neurite to become an axon. In a follow-up study, Shelly et al. examined the effects of Semaphorin3A (Sema3A), a secreted molecule that guides axon/dendrites growth and neuronal migration (Shelly et al., 2011). Here, the researchers utilized the biosensors cGES-DE5, ICUE, and AKAR to monitor the effects of Sema3a and BDNF on cAMP and cGMP. Bath application of Sema3A led to a decrease in the levels of cAMP and PKA activity and an increase in cGMP. Bath application of BDNF led to the opposite changes in cAMP, PKA, and cGMP. Furthermore, blocking soluble guanylyl cyclase (sGC) and PKG with small molecule inhibitors prevented the increase in cGMP by Sema3A, indicating that Sema3A exerts its effects via PKG regulation of sGC. The same compounds prevented the Sema3A induced decrease in cAMP. These results suggest that Sema3A and BDNF exert opposing actions on axon-dendrite differentiation mediated through reciprocal regulation of cyclic nucleotides, consistent with their previously reported findings (Shelly et al., 2010). This study revealed Sema3a’s role as a polarizing factor which favors the differentiation of neurites to dendrites while suppressing axon formation in cultured hippocampal neurons.

### Growth

Cyclic adenosine monophosphate probes can be used to dissect the specific contributions of cAMP modulating GPCRs to physiological changes like axon growth. ICUE3 was recently used to investigate the impact of ionotropic and metabotropic purinergic receptor signaling on axon elongation (del Puerto et al., 2012). Metabotropic P2Y receptors are activated by ADP, whereas the ionotropic P2X receptors are activated by ATP. By modulating purinergic signaling pathways in cultured hippocampal neurons, the authors found that P2Y1 enhances axonal elongation while P2Y13 and P2X7 halt axonal elongation. Addition of ADP or a P2X7 antagonist increased cAMP levels in the distal region of the axon as reported by ICUE3. Concurrent application of an AC5 inhibitor prevented the cAMP increase. Therefore, the purinergic receptors regulate cAMP levels through AC5 and regulate axonal elongation triggered by neurotrophic factors.

### Guidance

Cyclic adenosine monophosphate is a second messenger that has long been appreciated in guiding axon elongation as the first step in neural circuit formation. The high spatial and temporal resolution of FRET based biosensors allows researchers to study transient cAMP signals in restricted areas of the neuron such as the growth cone, a structure at the tip of the growing axon that regulates axonal growth and pathfinding. Epac2-camps was used extensively in a study that investigated the spatial and temporal dynamic interactions between cAMP and calcium within the axonal growth cone and its filopodia (Nicol et al., 2011). By fusing the sensor to an N-terminal plasma membrane localization signal (pm-Epac2-camps), the researchers were able to reduce differences in fluorescence intensity between the filopodia and growth cone center allowing for direct comparison of FRET signals. First, the authors showed that bath application of the axon guidance molecule Netrin-1 induces cAMP and calcium transients with similar kinetics. Next, by modulating AC, they were able to show that Netrin-1 induced calcium transients in growth cone filopodia are driven by transient elevation of cAMP. The growth cone center, on the other hand, was shown to have a delayed and prolonged calcium driven cAMP increase as compared to the filopodia. Therefore, the cAMP in the growth cone center is downstream of calcium, opposite that of the filopodia.

### Signaling and excitability

Genetically encoded biosensors have provided direct evidence for signaling compartmentalization in neurons. For example, the AKAR biosensor targeted to nuclear and cytosolic compartments revealed that PKA signaling within the nucleus of thalamic intralaminar neurons is delayed four-fold as compared to the cytosol (Gervasi et al., 2007). This data is consistent with the view that PKA signals propagate slowly to the nucleus via diffusion of the PKA catalytic subunit (Harootunian et al., 1993). Additionally, the thin dendrites of mouse cortical neurons exhibit larger cAMP and PKA responses than the bulk cytosol (Castro et al., 2010). This data corresponds well to modeling predictions of stronger cAMP and PKA signals in regions which have higher surface area to volume ratios (Neves, 2012). Castro et al. further compared cAMP/PKA responses in mouse brain slices triggered by dopamine D1 receptors in the cortex and the striatum (Castro et al., 2013). Biosensor imaging in pyramidal cortical neurons and striatal medium spiny neurons (MSNs) showed that cAMP/PKA response was stronger, faster, and longer lasting in the striatum than the cortex. They attributed this to more active PDE4 in the cortex, stronger AC activity in the striatum, and the phosphatase inhibitor DARPP-32 in the striatum which prolong the effects of PKA substrate phosphorylation. Thus, the PKA signaling cascade exhibits differential integration of upstream modulators within different brain areas and cell types.

Signaling crosstalks can also be dissected using fluorescent biosensors. Polito et al. used Cygnet2 and Epac-S^H150^ to monitor cGMP and cAMP, respectively in medium spiny neurons of the striatum (Polito et al., 2013). They studied the NO response in striatonigral and striatopallidal MSNs and found that it was partially controlled by PDE2. D1 and D2 MSNs were found to have different transient cAMP responses to brief Fsk stimulation. PDE2 activation by cGMP prevented these cAMP responses and was magnified at the level of PKA activity as visualized by the AKAR3 biosensor. Therefore, PDE2 is a critical effector of NO and modulates the post-synaptic response of MSNs to dopaminergic transmission.

The application of fluorescent biosensors has also provided new insights into signaling mechanisms. It is known that dopamine D1 receptors undergo rapid endocytosis following agonist induced activation. In another recent study, researchers from the von Zastrow lab set out to investigate the functional significance of dopamine receptor endocytosis using the Epac1-camps biosensor in cultured striatal neurons (Kotowski et al., 2011). In this study, internalization of a pH-sensitive fluorescently tagged D1R was tightly coupled (<1 min.) to an increase in cAMP upon stimulation with the D1R agonist SKF81297. The researchers then showed there was a causal relationship between internalization and cAMP accumulation by treating cultured striatal neurons with dynasore, a dynamin inhibitor to block endocytosis. Dynasore reduced the effect of dopamine mediated cAMP elevation indicating that D1R activation and endocytosis is responsible for cellular cAMP accumulation. This study showed that D1R endocytosis supports rapid dopaminergic neurotransmission through the early endocytic pathway.

### Plasticity

The use of fluorescent biosensors in the field of neuroscience can lead to discoveries of previously undefined roles for signal transduction pathways. The synapse is the primary site of neurotransmission which undergoes a great deal of remodeling known as synaptic plasticity. The cAMP/PKA pathway regulates changes at the pre- and post-synapse through PKA phosphorylation of synaptic membrane components as well as cAMP responsive element binding protein (CREB) mediated gene transcription (Kandel, 2012). Modulation of normal synaptic processes leads to changes in cognition and behavior such as aggression, fear, anxiety, and learning and memory (Wallace et al., 2009; Liu et al., 2014). In 1991, a WD repeat actin binding protein called coronin 1 was identified in D. *discoideum* (de Hostos et al., 1991). Absence of coronin 1 in transgenic mice lead to defects in synaptic plasticity and behavior. Recently, Jayachandran et al. investigated the mechanism by which coronin 1 exerts its effects on the nervous system (Jayachandran et al., 2014). Investigation into the cellular localization of coronin 1 found that it localizes to excitatory synapses and not inhibitory synapses. Furthermore, PKA-dependent pre-synaptic LTP was absent in coronin 1 null mice indicating a defect in PKA signaling. Other experiments examining PKA phosphorylation of CREB and cAMP related physiological changes further suggested a change in cAMP/PKA signaling in coronin 1 null mice. To directly measure how cAMP is affected by coronin 1, the researchers used ICUE3 to monitor cAMP production in the coronin 1 deficient cell line Mel JuSo. Treatment with isoproterenol led to a minimal increase in cAMP whereas co-transfection of ICUE3 with a coronin 1 expression plasmid produced a robust increase in the cyan to yellow emission ratio. By combining the above data and data from protein-protein interaction experiments, the researchers concluded that coronin 1 potentiates cAMP/PKA signaling by positively interacting with the G-protein subunit Gαs.

## Advances in the field

### Brain slice and live-animal imaging

From shedding light on the kinetics of neuromodulatory signaling events to uncovering new aspects of signal transduction, fluorescent biosensors have been an invaluable tool for neuroscience research. Over the past decade, FRET-based biosensors have been used to dissect the intricacies of cyclic nucleotide signaling in the nervous system with unprecedented detail. Second messenger signaling is now being studied in more physiologically relevant samples like brain slices and transgenic animals with FRET-based biosensors (Calebiro et al., 2009; Thunemann et al., 2013). Polito and colleagues recently published a thorough protocol for imaging biosensors like AKAR in brain slices (Polito et al., 2014). These techniques are more demanding as they require intensive sample preparation and advanced equipment to preserve the spatial resolution afforded by single cell imaging experiments. The development of far-red and infrared biosensors will give researchers the ability to probe the biochemistry of cyclic nucleotides in deep tissues difficult to reach with current imaging methods (Shcherbakova and Verkhusha, 2013). Advances in microscopy tools are also an integral part of live brain imaging research. One such tool is fibered fluorescence microscopy that allows for a tissue imaging depth of up to 6 mm (Vincent et al., 2006). Using this technique, Vincent et al. were able to image peripheral nerve regeneration and calcium dynamics in central nervous nuclei of an anesthetized mouse. Although it has not yet been demonstrated, it is likely that the next wave of *in vivo* brain imaging will include studies of cyclic nucleotide signaling dynamics in live animals.

### Perturbation of cyclic nucleotide signaling in living cells

In addition to monitoring the biochemistry underlying neuronal activity, researchers are constantly developing innovative tools to perturb signaling molecules. One of these tools has been developed over the past decade in order to control the activity of neurons with light (Boyden et al., 2005). The technique is based on the light-driven ion channel called channelrhodopsin which can be genetically encoded, expressed and manipulated with light to depolarize or silence individual or groups of neurons. Optogenetics was a great leap forward in neuroscience allowing researchers to map circuits in brain slices and control the neural activity of transgenic animals. Similar tools have been developed to perturb cyclic nucleotide signaling. Light driven modulation of cyclic nucleotides began with the development of caged molecules that are activated through a photolysis reaction (Nargeot et al., 1983). Dimethoxy nitrobenzyl caged-cAMP was used in combination with AKAR in several different studies (Castro et al., 2013). Coumarinylmethyl caged cNMP derivatives developed over the past decade are more efficiently released under nondamaging light conditions (>360 nm wavelength). (Hagen et al., 2001; Geißler et al., 2003). New bis-carboxymethyl variants can allow for one and two-photon flash uncaging and could provide cyclic nucleotide release within deep tissue preparations (Hagen et al., 2005).

Neuronal cyclic nucleotide research has benefited from the implementation of genetically encodable photoactivatable adenylyl cyclase enzymes (PAC). This valuable addition to the toolbox allows for active user-controlled manipulation of cAMP and cGMP levels in specific locations within the cell. These blue-light (450 nm wavelength) sensitive nucleotidyl cyclases respond to light via conformational changes in the light-oxygen-voltage (LOV) or sensors of blue light using FAD (BLUF) photoreceptor protein domains. EuPAC from *Euglena gracilis* was the first to have a demonstrated PAC activity. It was used to show for example that focal stimulation of intracellular cAMP can steer the growth of *Xenopus* spinal commissural axons (Nicol et al., 2011). However, it suffered from high dark activity and poor heterologous cell expression due to its large size (Iseki et al., 2002). bPAC, also known as BlaC, was discovered in *Beggiatoa* sp. It was significantly smaller than EuPAC with low dark state activity which could be tuned by manipulating the level of expression (Ryu et al., 2010; Stierl et al., 2011). Stierl et al. used bPAC to successfully induce activity in cultured hippocampal neurons co-transfected with cyclic nucleotide gated ion channel. Ryu et al. further engineered it into a photoactivatable guanylyl cyclase, BlaG, by introducing three mutations into the region responsible for substrate binding. BlaG is the first photoactivatable guanylyl cyclase. More recently, mPAC was discovered in *Microcoleus chthonoplastes* (Raffelberg et al., 2013). This enzyme uses a LOV domain rather than the BLUF domain as in EuPAC and bPAC and has a greater AC activity in both the dark and light-activated state. The *in vivo* application of the aforementioned PACs is limited by the low tissue penetrance of the blue light needed to activate them. To increase tissue penetration, researchers developed a near-infrared adenylyl cyclase (IlaC) by fusing a bacteriophytochrome BphG1 to a bacterial adenylate cyclase CyaB1. IlaC was expressed in *Caenorhabditis elegans* cholinergic neurons and was shown to induce locomotion with exposure to red light (650 nm) as measured by the number of body bends per minute (Ryu et al., 2014). Given the spectral separation, IlaC opens up the possibility for PACs to be combined with biosensors for real-time manipulation and monitoring of cAMP and cGMP signaling pathways.

### Computational modeling and simulation

A fundamental question in cyclic nucleotide research is the maintenance of specific signaling in the midst of multiple inputs and outputs. For example, within neurons there is substantial cross-regulation of cAMP, cGMP and Ca^2+^. How do multiple signaling pathways interact to form a coherent output instead of chaos? Despite the advances in monitoring and manipulating cyclic nucleotides, we still do not have a clear understanding and additional tools such as mathematic modeling are needed to understand the intricacies of second messenger crosstalk and compartmentation (Saucerman et al., 2014). Significant efforts by different laboratories have been directed towards building mechanistic models to drive research hypotheses. One such study has started to reveal how a neuron’s cell shape, topological distribution of biological components, and enzyme kinetics allows a cAMP/PKA microdomain to translate into a gradient of MAPK activity (Neves et al., 2008). The use of genetically encoded biosensors for imaging cyclic nucleotide signaling dynamics, either one at a time or better yet, in a co-imaging mode for multiple parameters, can help obtain more quantitative information, thereby facilitating model development and testing (Ni et al., 2011). Computational modeling efforts are aided by modeling software such as NeuroRD. These programs allow integration and simulation of structural data, enzyme kinetics, and diffusion rates for signaling networks. A guide to using NeuroRD is available with detailed information on developing and analyzing computational models of neuronal signaling networks (Blackwell et al., 2013). This tool has been used to demonstrate the role of PDE4D in maintaining subcellular cAMP microdomains with the requirements of PDE4D being anchored in the bulk cytosol and regulated by PKA phosphorylation (Oliveira et al., 2010). Similarly, stochastic reaction-diffusion models of dopamine signaling in the dendrites have demonstrated the importance of subcellular PKA colocalization with other components of the pathway to determine spatial signaling specificity (Oliveira et al., 2012).

With the multitude of neuromodulatory inputs affecting cyclic nucleotide levels, signaling information needs to be encoded specifically to produce specific cellular responses. Encoding can occur on spatial, temporal, and digital levels using molecular distribution, enzyme kinetics, and signal thresholding to elicit various physiological outputs from cAMP and cGMP signal transduction (Rich et al., 2014). Our understanding of cyclic nucleotide signaling within the context of neuromodulation continues to grow, facilitated by advances in biosensor and imaging technology. An interdisciplinary approach combining experimental and computational strategies may pave the way for future discoveries.

## Conflict of interest statement

The authors declare that the research was conducted in the absence of any commercial or financial relationships that could be construed as a potential conflict of interest.

## Abbreviations

cyclic nucleotide binding domain: CNBD
Dopamine 1 receptor: D1R
light-oxygen-voltage: LOV
sensors of blue light using FAD: BLUF.
